# Evolution of healthcare-associated infection surveillance in England: initiatives, implementation and opportunities for innovation

**DOI:** 10.1186/1753-6561-5-S6-P229

**Published:** 2011-06-29

**Authors:** R Freeman

**Affiliations:** 1 Infectious Diseases and Immunity, Imperial College London, London, UK

## Introduction / objectives

Surveillance data needs to be capable of; identifying clusters; tracking trends; assessing the effectiveness of interventions; and providing timely results. This research assesses whether this is the case for healthcare-associated infection (HCAI) and antimicrobial resistance (AMR) surveillance systems in England, and endeavours to understand why in certain areas surveillance has not been more successful.

## Methods

A systematic search of the literature was carried out. Original search results identified additional Government, committee and conference reports and guidelines.

## Results

Surveillance of HCAIs and AMR has developed over time in response to numerous stimuli. The implementation of novel surveillance systems are often in response to issues identified through passive surveillance and research. Evidence of rising infection or resistance rates have caught the attention of the Government (and the media) occasionally leading to enhanced surveillance of specific infections to monitor progress towards targets. Several independent studies have concluded that post-discharge surveillance after surgery is required to provide more accurate surgical site infection rates. Funding for surveillance programmes has come from various sources including the pharmaceutical industry, particularly in AMR surveillance.

## Conclusion

Some surveillance schemes have been successfully implemented, partially fulfilling criteria. However, many clinical areas have yet to see advances in HCAI surveillance specific to their area and are reliant upon generic passive surveillance systems. Future development of surveillance systems to rapidly identify clusters and outbreaks, and patients at increased risk of acquiring infection will ensure that control measures are best directed to prevent further cases occurring.

## Disclosure of interest

None declared.

